# Role of cellular senescence in inflammation and regeneration

**DOI:** 10.1186/s41232-024-00342-5

**Published:** 2024-06-03

**Authors:** Yuki Saito, Sena Yamamoto, Takako S. Chikenji

**Affiliations:** 1 Department of Anatomy, Sapporo Medical University School of Medicine, Sapporo, 060-8556 Japan; 2 Graduate School of Health Sciences, Hokkaido University, Sapporo, 060-0812 Japan

**Keywords:** Cellular senescence, Senescence-associated secretory phenotype (SASP), Cell cycle arrest, Tissue remodeling, Aging, Fibrosis

## Abstract

Cellular senescence is the state in which cells undergo irreversible cell cycle arrest and acquire diverse phenotypes. It has been linked to chronic inflammation and fibrosis in various organs as well as to individual aging. Therefore, eliminating senescent cells has emerged as a potential target for extending healthy lifespans. Cellular senescence plays a beneficial role in many biological processes, including embryonic development, wound healing, and tissue regeneration, which is mediated by the activation of stem cells. Therefore, a comprehensive understanding of cellular senescence, including both its beneficial and detrimental effects, is critical for developing safe and effective treatment strategies to target senescent cells. This review provides an overview of the biological and pathological roles of cellular senescence, with a particular focus on its beneficial or detrimental functions among its various roles.

## Introduction

Cellular senescence was first described approximately 60 years ago by Hayflick and Moorhead [1]. They observed that normal human fibroblasts have a finite proliferative capacity in culture and termed the cell cycle arrest at the exhaustion of this capacity “replicative senescence;” the word “senescence” is derived from the Latin word *senex*, meaning “old.” Later, it was discovered that cellular senescence occurs because of telomere shortening, which leads to chromosomal instability and is considered a tumor suppressor mechanism [2, 3]. Subsequent research has uncovered the vital role of cellular senescence in various physiological processes beyond its tumor-suppressive functions, such as wound healing [4, 5], embryonic development [6], and tissue repair and regeneration. Cellular senescence contributes to aging and age-related diseases [7]. Senescent cells are metabolically active and secrete a variety of factors, including inflammatory cytokines and chemokines. These are collectively termed the senescence-associated secretory phenotype (SASP), which can induce both chronic inflammation (detrimental effects of cellular senescence) and tissue remodeling (beneficial effects of cellular senescence) [8–12]. However, both forms of senescence show expression of tumor suppressor genes (p53, p16^INK4a^, p21^WAF1/Cip1^) and senescence-associated-β-galactosidase (SA-β-gal), which are used as markers of cellular senescence. However, the characteristics and features of each type of senescence have not yet been clearly distinguished.

This review focuses on the role of senescent cells in both tissue regeneration and inflammation while considering both perspectives of beneficial and detrimental effects of cellular senescence.

## Triggers and features of senescence

Cellular senescence is triggered by various physiological and pathological stressors and leads to irreversible cell cycle arrest [10, 13–15]. Cells undergo senescence in response to various stressors, including telomere shortening [16, 17], oncogene activation [18], radiation [19], high levels of reactive oxygen species (ROS) [20], mitochondrial dysfunction [21], inflammatory cytokine and chemokines [22], mechanical stress [23], protein aggregation [24], failure of protein removal due to diminished autophagy [25], ribosome stress[26, 27], nutrient imbalance [10], inflammatory cytokines, and growth factors [28, 29]. These different types of stressors induce different types of senescence, such as replicative senescence (RS), oncogene-induced senescence (OIS), therapy-induced senescence (TIS), stress-induced senescence (SIS), mitochondria dysfunction-induced senescence (MiDAS), and immunologically-induced senescence (IIS). RS is a phenomenon characterized by telomere shortening, which results in chromosomal instability and triggers DNA damage [30, 31]. DNA damage is often reported as a common underlying cause of senescence. It is primarily reported in the form of DNA double-strand breaks, which activate the DNA damage response (DDR) [32]. DDR factors accumulate at sites of DNA damage and contribute to cell cycle arrest by phosphorylating histone H2AX (γH2AX) and exhibiting nuclear foci such as mediator of DNA damage checkpoint 1(MDC1) and p53 binding protein 1 (53BP1). In response to persistent DNA damage, DDR signaling is prolonged, resulting in the activation of ataxia telangiectasia mutated (ATM), ATM and Rad3-related (ATR), checkpoint kinase (CHK) 1, and CHK2. This ultimately leads to cellular senescence via activation of p53 and the subsequent cyclin-dependent kinase inhibitor p21^WAF1/Cip1^, resulting in cell cycle arrest [33]. OIS is induced by the expression of oncogenes such as *NRAS*^*G12V*^ and *BRAF*^*V600E*^, which mediate cell cycle arrest via p21^WAF1/Cip1^ and p16^INK4a^, and operate as a cell-intrinsic tumor-suppressive mechanism [3, 31, 34]. TIS is induced by cancer treatments such as chemotherapy and radiation, which trigger DDR [34]. SIS is typically characterized as telomere-independent cellular senescence that originates in response to chemical or physical stressors inducing oxidative stress and DNA damage [35, 36]. MiDAS is mitochondrial damage that triggers senescence with a distinct secretory phenotype that lacks IL-1-dependent inflammation [37]. IIS is induced by excessive pro-inflammatory factors, particularly IL-17. It demonstrates a distinct secretory phenotype that alters WNT signaling and extracellular matrix remodeling [22].

Senescence is a cellular state that can be induced by different stimuli as described above, and a major common feature of senescent cells is stable cell cycle arrest and SASP [10]. The primary mediators of cell cycle arrest in senescent cells are p21 ^WAF1/Cip1^ and p16^INK4a^, which inhibit the activity of cyclin-dependent kinases (CDKs), resulting in the repression of E2F transcription factors and their target genes, including those encoding cyclin E and cyclin D, via hypophosphorylated retinoblastoma (Rb), p107, and p130. This process leads to the arrest of the cell cycle in the G1 phase [34]. The SASP is the primary mediator of the paracrine effects of senescent cells. It demonstrates a wide range of both local and systemic biological impacts [9, 11, 12, 38]. The secretion is regulated by pathways that involve nuclear factor kappa-light-chain-enhancer of activated B cells (NF-κB), p38 mitogen-activated protein kinase (p38MAPK), mammalian target of rapamycin (mTOR), CCAAT-enhancer-binding protein β (C/EBPβ), and cyclic GMP–AMP synthase (cGAS)–stimulator of interferon genes (STING), and the main components of SASP include pro-inflammatory cytokines (for example, interleukin[IL]-1β, IL-6, and IL-8), chemokines (for example, C–C motif chemokine ligand [CCL]2, CCL5, and C-X-C motif chemokine ligand [CXCL]1), growth factors (for example, transforming growth factor-β [TGF-β], epidermal growth factor [EGF], and growth differentiation factor [GDF]15), proteases (for example, matrix metallopeptidase [MMP]1 and MMP3), bioactive lipids (for example, prostaglandins and leukotrienes), extracellular matrix components, and non-coding nucleic acids (for example, microRNA and cytoplasmic chromatin DNA fragments) [9, 11, 39–41].

Hence, while the triggers and characteristics of senescence are becoming clear, specific markers to detect senescent cells, especially under in vivo conditions, have not been fully identified [10]. Currently, the combination of multiple markers is recommended for the identification of senescent cells [10, 42]. The first step of the proposed workflow is assessing SA-β-gal activity (X-Gal or the fluorescent probes C12FDG, SPiDER-β-Gal, and DDAO galactoside) or lipofuscin accumulation (Sudan Black B [SBB] or SBB analog [GL13]). SA-β-gal activity is one of the most widely used markers of senescence; however, it has been observed in non-senescent cells and macrophages as well, which reduces its reliability as a standalone marker for senescent cells [43, 44]. Second, co-staining with senescence-inducing factors like p16^INK4a^ and p21^WAF1/Cip1^ and the absence of cell proliferation indicators, including Ki-67, proliferating cell nuclear antigen (PCNA) expression, or EdU/BrdU incorporation, is required [10, 42]. Recently, a new detection method for p16^INK4A^, which has been challenging to detect in mouse tissues, has been developed and holds promise for utility [45]. Third, markers for specific types of senescence, including γH2AX, indicate sustained DDR, SASP expression, and anti-apoptotic factors, is recommended [10, 42]. The utility of mass cytometry by time-of-flight (CyTOF) in such multiparametric detection of senescent cells has also been demonstrated, and senescence CyTOF antibody panels have been developed to detect senescent cells in mice and humans. The p16^INK4a^-high cells identified in the panel were characterized by cell cycle arrest, apoptosis resistance, and enriched in DDR [46]. Furthermore, with the development of transcriptome analysis, especially single-cell RNA-seq analysis, it is desirable to develop a method to identify senescent cells at the transcriptome level. To address this need, based on an extensive review of the literature, a panel of 125 genes was developed as the SenMayo senescence gene set [47]. Additionally, a machine learning program senescent cell identification (SenCID) has been developed, which was trained on 602 samples derived from 52 senescence transcriptome datasets across 30 cell types [48].

## Bright and dark sides of senescent cells

**Fig. 1:**
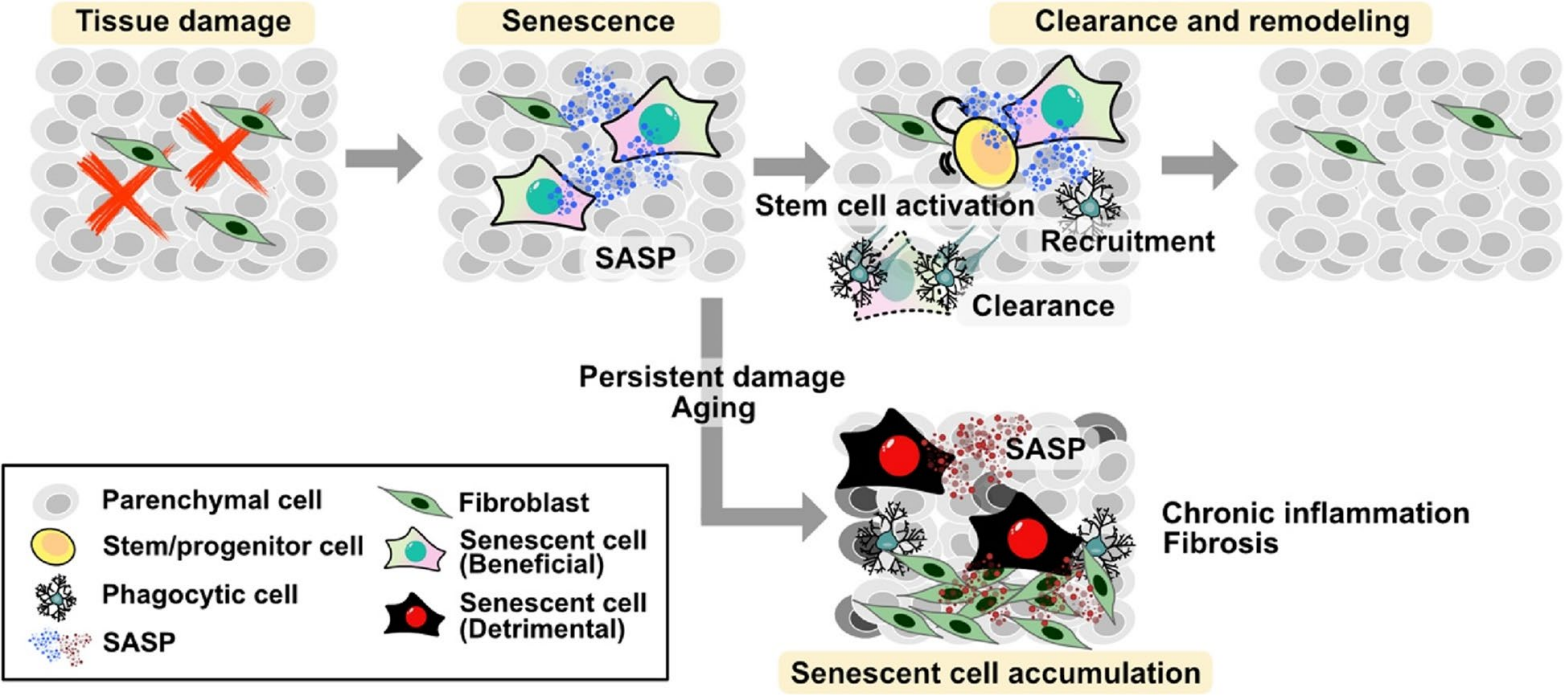
Bright and dark sides of senescent cells. Tissue damage-induced cellular senescence recruits immune cells, especially phagocytic cells, by secreting senescence-associated secretory phenotypes (SASP), which also promotes tissue regeneration by stimulating the self-renewal and differentiation of tissue-resident stem/progenitor cells. The remodeling process is completed when senescent cells are cleared by phagocytic cells, resulting in a senescence-clearance-remodeling sequence. However, this sequence can be disrupted by persistent damage or aging, leading to the accumulation of detrimental effects on senescent cells and the induction of chronic inflammation and fibrosis via the persistence of SASP

Recent studies have shed light on the features and physiological roles of senescent cells, demonstrating that cellular senescence is not merely a state of cell cycle arrest, but rather a dynamic process that influences other biological activities. Cellular senescence plays roles in diverse processes, including embryonic development, wound healing, tissue repair, regeneration, cancer, aging, age-related diseases, and chronic inflammatory diseases. Senescence triggers a process of tissue remodeling; the beneficial effects of senescent cells involve the promotion of the recruitment of immune cells, especially phagocytic cells, and the activation of tissue-resident stem/progenitor cells through the release of SASP, resulting in tissue regeneration [13, 49] (Fig. 1). However, this state can be disrupted by persistent damage or aging. In these instances, detrimental effects of senescent cells accumulate and induce chronic inflammation and fibrosis via persistent SASP [13] (Fig. 1). In certain situations, these seemingly contradictory roles can be difficult to reconcile, making this a confusing and still not fully elucidated phenomenon.

## Cellular senescence in aging and age-related disease

From the 1990s to the 2000s, several studies showed that senescent cells increase in abundance with aging and age-related diseases in vivo [50–55]; however, the causal relationship between cellular senescence and aging or age-related diseases remains unclear. In 2011, a transgenic system called INK-ATTAC mice was used to provide additional evidence supporting the significant role of senescent cells in aging and disease [56]. This model allows the targeted removal of senescent cells by combining the p16^Ink4a^ promoter with an FKBP-caspase-8 suicide transgene, which activates caspase-8 and induces apoptosis, specifically in p16^Ink4a^-high senescent cells, upon the administration of an FKBP dimerizer. The study showed that p16^INK4a+^ cells were increased in the adipose tissue, skeletal muscle, and eyes of BubR1^H/H^-progeria mice, and selective elimination of p16^INK4a+^ cells delayed the onset of age-related phenotypes, including muscle atrophy, lordokyphosis, cataracts, and lipodystrophy [56]. Subsequently, the research group employed naturally aged INK-ATTAC mice to demonstrate whether senescent cells are responsible for age-related phenotypes and healthy lifespans [57]. They showed that the selective elimination of p16^INK4a+^ cells increased the median lifespan of both male and female mice, as indicated by reduced glomerulosclerosis and cardiomyocyte hypertrophy [57]. The INK-ATTAC mouse model comprises a 2,617-bp fragment of the *p16*^*INK4a*^ gene promoter. The removal of p16-expressing cells has been proposed to result in life extension in mice. However, there is a concern about whether the reporter constructs with a part of the p16 genomic sequence fully resembles endogenous p16 gene expression. Since *Cdkn2a* gene expression is thought to be accompanied by massive chromatin structural changes [58], it is essential to carefully examine whether the effects observed in these mice could be attributed only to the elimination of senescent cells. This is further supported by the fact that some p16-expressing cells are not efficiently removed by the INK-ATTAC system in several tissues, including the liver, colon, and T lymphocytes [57].

Cellular senescence not only contributes to the aging process but also actively participates in the development and progression of various age-related diseases. Senescent cell accumulation is frequently observed at sites associated with the pathogenesis of several prominent age-related chronic diseases, such as Alzheimer’s disease, cardiovascular diseases, osteoporosis, diabetes, renal disease, and liver cirrhosis [59–66]. Notably, transplanting a small number of senescent cells into young, healthy animals recapitulates age-related physical impairments, induces osteoarthritis, and increases mortality rates [67, 68]. Genetic elimination of p16^Ink4a^-high senescent cells in INK-ATTAC mouse models has shown promising results in preventing or mitigating various diseases, such as osteoporosis, frailty, atherosclerosis, hepatic steatosis, osteoarthritis, idiopathic pulmonary fibrosis, obesity-induced anxiety, tau-mediated neurodegenerative disease, and type 2 diabetes mellitus/metabolic dysfunction [56, 65, 69–71]. Consistent with these findings, investigations employing a transgenic p16-3MR (trimodality reporter) mouse model expressing luciferase and red fluorescent protein reporters along with herpes simplex virus-1 thymidine kinase (HSV-TK) have demonstrated that genetic depletion of p16^Ink4a^-expressing senescent cells using ganciclovir (GCV) as an apoptosis inducer can alleviate various age-related dysfunctions [70, 72]. In the p16-3MR mouse, a bacterial artificial chromosome was engineered to contain approximately 50 kb fragment comprising the murine *p16*^*INK4A*^ locus. The p16^INK4A^ promoter drives 3MR expression, and HSV-TK facilitates killing by GCV, a nucleoside analog that inhibits DNA synthesis. It is considered that senescent cells exit the cell cycle and stop replicating their genomic DNA, resulting in low affinity for TK in senescent cells. However, the authors explained that HSV-TK converts GCV into a toxic DNA chain terminator. In non-dividing senescent cells, GCV fragments mitochondrial DNA, causing death by apoptosis [73].

In another model, researchers targeted p19^ARF^ (14ARF mouse homolog), which expresses a diphtheria toxin receptor, along with the bioluminescent enzyme luciferase under the ARF promoter. This model allows the induction of apoptosis in p19ARF-positive senescent cells by administering the diphtheria toxin [74]. The results demonstrated that eliminating p19^ARF^-positive senescent cells in the lung tissue improved age-related decreases in lung function and reversed the aging-associated gene expression profile [74].

Recently, p16-Cre and p21-Cre transgenic mouse models were developed, and the use of Cre-inducible expression of the DTR system showed that the elimination of p16- or p21-positive cells inhibited SASP expression [75–77]. Grosse et al. developed a p16-Cre knockin mouse model, which integrated a targeting cassette in the last exon of the endogenous p16^INK4a^ gene [78]. This allowed the preservation of most of the p16^INK4a^ genomic sequence and binding sites for many regulators of p16 transcription. The researchers integrated Cre recombinase, TK, and tdTomato (a fluorescent reporter) at the end of the third exon by fusing it with p16^INK4a^ mRNA via self-cleaving peptide sequences that would produce separate proteins after translation. However, the introduction of a fluorescent reporter in the targeting cassette was found to be impractical due to the low expression of p16 mRNA in vivo, and a similar low level of p16-driven fluorescence was reported in other studies [4]. The p16-Cre knockin mice were crossed with Rosa26-mTmG mice. Most p16^high^ cells were vascular endothelial cells, mostly found in liver sinusoids in twelve-month-old mice. In contrast, mice engineered with p16-CreETR2/Rosa26-DTA (p16-expressing cells were removed in 1.5-year-old mice) showed disrupted blood tissue barriers and subsequent liver, kidney, heart, and lung damage [78].

Omori et al. also generated p16-Cre ERT2-tdTomato mice with cells showing high p16^INK4a^ expression in vivo [77]. They tested various Cre-containing cassette genes and found that integration of a Cre ERT2 and a neo-resistant gene markedly resulted in higher labeling efficacy in the case of the Ink4a locus, as characterized in another report [79]. The findings were attributed presumably to the fact that a retained neo-resistant cassette can act as a local enhancer to augment reporter expression without compromising tissue reporter fidelity [79, 80]. The authors also crossed p16-Cre ERT2 mice with Rosa26-DTR-tdTomato mice, which led to the ablation of p16-expressing cells. Nonalcoholic steatohepatitis (NASH) was induced by a high-fat diet administered for 6 weeks in p16-CreERT2 DTR-tdTomato mice, and elimination of p16-expressing cells ameliorated steatosis and inflammation in the liver of the mice with NASH [77].

The use of a transgenic cell cycle maker-expressing senescent cell removal system in mice has facilitated studies on senescent cells in aging and age-related diseases.

Accumulation of senescent cells and chronic inflammation resulting from SASP secretion are mechanisms of senescent cell-mediated aging and age-related diseases. Although senescent cells are damaged by DNA breaks and exhibit increased ROS production, they do not undergo apoptosis. Instead, they activate pro-survival pathways, known as senescent cell anti-apoptotic pathways (SCAPs), while downregulating key apoptotic mediators [81–85]. Transcriptome analysis of senescent and non-senescent human cells initially identified SCAPs, which were subsequently confirmed by RNA interference experiments [83]. These pathways include the B-cell/CLL lymphoma (BCL)-2 family, phosphoinositide 3-kinase (PI3K)-Akt, p53-p21-serpines, hypoxia inducible factor (HIF)-1, and heat shock protein (HSP)90 [82, 83]. They confirmed that dasatinib, a tyrosine kinase inhibitor, and quercetin, a flavonoid, which inhibit the PI3K-AKT signaling, decreased senescent cells, ameliorated aging-related phenotypes, and extended the health span of progeroid Ercc1−/Δ mice [83]. Recently, single-cell transcriptomics revealed that MCL-1 also contributes to the anti-apoptotic features of senescent cells and that MCL-1-positive cells have higher SASP expression than BCL-2-positive senescent cells [85].

The accumulation of senescent cells in aging tissues, coupled with the detrimental impact of the SASP, plays a significant role in driving the aging process and the development of age-related diseases [82]. In contrast, some mouse models suggest that the removal of senescent cells may worsen healthy life expectancy [78]. Further examination is required to delineate the potential of targeting senescent cells to extend healthy life expectancy.

## Cellular senescence in developmental disorders, genetic disorders, and autoimmune chronic inflammatory diseases

In addition to age-related diseases, developmental, genetic, and autoimmune diseases are affected by senescent cells regardless of age. Some examples include neurodevelopmental defects [86–88], Duchenne muscular dystrophy (DMD) [89], and autoimmune diseases [90–92]. Drug use during pregnancy and maternal diabetes are major non-genetic factors in neurodevelopmental defects. Valproic acid exposure- or diabetes-meditated mouse models of neurodevelopmental defects showed increased senescent neuroepithelial cell abundance and SASP expression [87, 88]. These studies also demonstrated that the knockout of cyclin-dependent kinase inhibitors or senomorphic rapamycin treatment rescued neurodevelopmental defects [87, 88]. According to another study, senescence plays a role in the neurodevelopmental pathogenesis of Down syndrome. Induced pluripotent stem cells (iPSC)-derived neural progenitor cells exhibit features of senescence in response to the triplication of chromosome 21, and senolytic drugs can alleviate the transcriptional, molecular, and cellular dysfunctions associated with Down syndrome [86]. DMD, another genetic disorder, also increases the number of senescent cells in the skeletal muscle of patients with DMD or DMD model rats; senolytic treatment prevented the loss of body weight and muscle strength in the model rats [89]. In autoimmune diseases, senescent neural cells and tubular epithelial cells are increased in the brain and kidneys of lupus model mice, and senolytic treatment decreased SASP expression and improved depression-like behavior and renal function [90, 91]. Furthermore, oral lichen planus, a chronic autoimmune oral mucosal disease, increased senescent mesenchymal cell abundance. Single-cell RNA-seq and cell-cell communication analyses showed that senescent mesenchymal cells significantly influenced CD8^+^ T cells and natural killer cells via CXCL12-CXCR4 signaling to develop a persistent inflammatory condition [92]. These reports indicate that senescent cells may be drivers of and promising therapeutic targets for various inflammatory diseases, in addition to aging.

## Cellular senescence in tissue regeneration

With regard to cellular senescence and its role in tissue regeneration, senescent cells with exceptional regenerative capacities are present in the amputated limbs of salamanders [93]. Furthermore, it has been suggested that the regeneration process is triggered by macrophage-mediated removal of senescent cells [93]. Recently, cellular senescence was shown to be induced in adult zebrafish fins following amputation. The peak of senescent cells occurred 8 days after amputation, and by 30 days after amputation, regeneration was complete. Furthermore, the removal of senescent cells with ABT263, an anti-apoptotic protein (BCL-2 family) whose expression is upregulated in senescent cells, decreased their regenerative ability [94]. Such a transient appearance of senescent cells has also been observed in tissue regeneration processes in neonatal mouse and zebrafish models of partial heart defects [95]. The study demonstrated that, although SA-β-gal positive senescent fibroblasts began to appear 3 days after cardiac apex resection in neonatal mouse pups, senescent cells were barely detectable on day 21 after resection when tissue regeneration was complete. In addition, fibroblast-specific deletion of Trp53 inhibited senescence induction, resulting in tissue fibrosis. Furthermore, the expression level of cellular communication network factor 1 (CCN1), a cysteine-rich protein component of the extracellular matrix (ECM) that inhibits fibrosis in skin wound healing by activating the Trp53 and p16^INK4a^ pathways, was found to be higher in the periapical region. To investigate its role, CCN1 was knocked down using an adeno-associated virus vector, which resulted in a reduction in the number of senescent cells 7 days after cardiac apex resection. Additionally, a decrease in the number of cardiomyocytes and an increase in fibroblast proliferation was observed, ultimately leading to impaired tissue regeneration and fibrosis [95]. This phenomenon was also observed in the livers of 2–3-month-old mice, indicating that stellate cells undergo cellular senescence within 2 days of resection following 2/3 hepatectomy. Following the administration of ABT263, liver regeneration was exacerbated, and it was shown that CCN1 plays a role in this tissue regeneration process [96].

In the skeletal muscle, platelet-derived growth factor receptor alpha (PDGFRα)^+^ fibro/adipogenic progenitors (FAPs) increased the expression of senescence and SASP factors, such as Cdkn2a, Trp53, and Il33, 2–3 days after muscle injury, and the senescent FAPs disappeared 14 days after injury when muscle regeneration occurred [49, 97]. In contrast, the accumulation of FAP in a mouse model of polymyositis (experimental autoimmune chronic myositis) reportedly results in fibrosis, with the expression levels of Cdkn2a and Trp53 being less prominent than those of FAP in acute inflammation [49, 97]. Next, to verify whether differences in the expression of senescence factors in FAPs affected muscle regeneration, FAPs from *Trp53* knockout and wild-type mice were isolated and transplanted into the skeletal muscle of another wild-type mouse; muscle damage was induced by barium chloride (BaCl_2_) after FAPs were implanted. Transplantation of FAPs from *Trp53*-knockout mice did not induce muscle regeneration but increased inflammation and induced fibrosis. Furthermore, when FAPs from mice with acute and chronic inflammation were isolated and co-cultured with muscle satellite cells, which are skeletal muscle stem cells, only senescent FAPs isolated from acute inflammation showed accelerated differentiation of muscle satellite cells. Interestingly, FAPs that increase the expression of senescence factors in acute inflammation also decrease the expression levels of T cell and macrophage checkpoints, such as programmed cell death ligand 1 (PD-L1) and CD47. Additionally, skeletal muscle undergoes muscle hypertrophy/regeneration upon exercise intervention, and exercise in wild-type mice transiently increases the expression of senescence factors in FAP, as observed during acute muscle injury. In contrast, in the mouse model of chronic myositis, the expression levels of senescence-related factors in the FAPs did not change after exercise. Rather, an increase was observed in the expression of nuclear factor-kappa B and α-smooth muscle actin, which contributed to fibrosis. Therefore, they hypothesized that inducing transient cellular senescence of FAP, similar to acute inflammation in FAP of chronic myositis, would promote muscle regeneration. They tested AICAR, an AMP-activated protein kinase (AMPK) activator, and reported that AICAR increased p16^INK4A^ expression in fibroblasts. Exercise intervention in AICAR-treated chronic myositis model mice induced FAP senescence; simultaneously, SASP factors changed to a pattern similar to that observed during acute inflammation, ultimately leading to muscle regeneration in chronic myositis [97]. This study relied on p16ink4a, p53, and SA-β-Gal to identify cell senescence but did not directly assess cell cycle arrest; therefore, further validation is needed to determine whether FAP promotes muscle regeneration by inducing senescence. Another study, which combined several approaches, including single-cell RNA-seq, spatial RNA-seq, and histological analysis, showed that senescent FAP and macrophages increased in the areas of muscle damage [98]. This study also showed that senolytics with ABT-263 decreased the number of muscle stem cells and inhibited muscle regeneration [98]. In contrast, Moiseeva et al. reported an increase in cellular senescent cell populations in FAPs, muscle stem cells, and macrophages, regardless of acute or chronic muscle injury or aging status, and genetic elimination of senescent cells using p16-3MR and pharmacological elimination using dasatinib and quercetin enhanced muscle regeneration [99]. In addition, the transplantation of SPiDER-β-Gal-positive senescent cells into skeletal muscle inhibited muscle regeneration after injury [99]. Considering the studies conducted thus far, it is evident that cellular senescence is a response to muscle injury; however, no single perspective has been established on the role of these senescent cells in facilitating or obstructing muscle regeneration. Additional research is warranted, as the findings may fluctuate depending on the specific cellular senescence markers employed in each study and the timing of cellular senescence induction or inhibition.

Recently, Reyes et al. created a highly sensitive fluorescent reporter of p16^INK4a^ expression in which an H2B-GFP cassette is inserted at the p16^INK4a^ locus to visualize transgenic mice (INKBRITE, INK4a H2B-GFP Reporter-In-Tandem) were generated to visualize p16^INK4a^ positive cells with GFP in vivo, and PDGFRα-positive fibroblasts expressing p16^INK4a^ are reportedly important for lung tissue regeneration [100]. CD45-positive immune cells or PDGFRα-positive fibroblasts were mainly detected according to their GFP signal in the lungs of healthy INKBRITE mice. p16^INK4a^-positive cells were surrounded by a laminin-positive basement membrane in the airway epithelium. Moreover, the p16^INK4a^-expressing PDGFRα-positive lung fibroblasts displayed characteristics of cellular senescence, including cell cycle arrest, polynucleation, DNA damage, and β-galactosidase expression. Following acute lung toxic naphthalene-induced injury to the lung epithelial tissue, the expression of SASP-related genes, including IL6 and epiregulin (EREG), increased in p16^INK4a^-positive fibroblasts. In vitro, studies demonstrated that p16^INK4a^-positive lung fibroblasts promote the proliferation of SCGB1A1-positive airway epithelial stem cell organoids. Furthermore, EREG, a component of the SASP, is a key mediator of damage-induced tissue regeneration in the airway epithelium induced by p16^INK4a^-positive fibroblasts. Knockdown of p16^INK4a^ reduced EREG expression levels in fibroblasts and inhibited lung epithelium regeneration [100].

Other studies have reported melanocytes where hair follicle stem cells gather in the upper part of hair follicles in nevus tissue. Furthermore, when melanocytes undergo senescence induced by the B-Raf proto-oncogene, serine/threonine kinase (BRAF) mutant genes and senescent melanocytes activate CD44-positive hair root stem cells and promote hair growth via osteopontin as a SASP [101]. These findings suggest that osteopontin secreted by melanocytes plays a role in this process. Additionally, studies have reported that senescent cells contribute to tissue regeneration by activating tissue stem cells, implying that these cells may play a role in maintaining biological tissue homeostasis.

These reports indicate that senescent cells may be drivers of and promising therapeutic targets for tissue regeneration.

## The role of cellular senescence in reprogramming

Recent developments in the field of cellular senescence have revealed their potential involvement in cellular reprogramming. Regarding the involvement of cellular senescence in reprogramming, expression of the Yamanaka 4 factors (OCT3/4, SOX2, KLF4, c-MYC [OSKM]) in mouse- and human-derived fibroblasts in vitro increased the expression of cellular senescence factors such as p16INK4a, SA-β-gal, p21, Senescence-related heterochromatin foci (SAHFs) in OSKM-induced cells, which results in the inhibition of reprogramming [102]. Silencing of *p53*, *p21* ^WAF1/Cip1^, *p16*^*INK4a*^, and other genes has been shown to increase reprogramming efficiency [102–104]. In contrast, in vivo, experiments using i4F mice, in which OSKM expression can be transiently induced to promote in vivo reprogramming, showed that SA-β-Gal-positive senescent cells were increased in the injured lung and skeletal muscle sites, while the number of NANOG-positive cells increased in the vicinity. Furthermore, *p16*^*INK4a*^ knockout or senolytic intervention by ABT-263 decreased reprogramming efficiency, indicating that senescent cells enhanced the reprogramming of neighboring cells [105, 106]. This cellular senescence-induced reprogramming is repeatedly mediated by secretion of the SASP factor IL-6, which activates and increases serine/threonine protein kinase, a Janus kinase signaling *cum* transcription activator target [105, 106].

*Hydractinia symbiolongicarpus* is a highly regenerative organism that can regenerate a new head within 3 days of decapitation. The population of pluripotent migratory stem cells, known as i-cells, is concentrated in the lower body part of the hydrozoan network and is originally absent in the head; no i-cells are known to be present immediately after decapitation. However, when transient SA-β-gal positive senescent cells emerge at the site of head amputation, they reprogram nearby somatic cells to generate de novo stem cells and regenerate the head [107]. Subsequently, senescent cells were eliminated by the senolytic ABT-263 or genetic inhibition of the CDKN1A-like gene (*Cdki1*), which impaired its reprogramming and regeneration. In contrast, the transient induction of senescence by photogenetic techniques reportedly increases the number of i-cells and regeneration [107].

## The role of cellular senescence in fibrosis inhibition

The development of tissue fibrosis is marked by excessive production of ECM due to the uncontrolled proliferation of fibroblasts and myofibroblasts in response to tissue damage [108]. Krizhanovsky et al. showed that senescence-activated hepatic stellate cells arise in the fibrotic region and are invariably located along the fibrotic scar in a carbon tetrachloride (CCl_4_)-induced liver fibrosis model [109]. To investigate whether senescence promotes or inhibits fibrosis, *p53* and *INK4A/ARF* double-knockout mice were treated with CCl_4_. The results showed an increase in the number of activated stellate cells and severe fibrosis [109]. These results suggest that senescence is involved in the inhibition of fibrosis and that senescent hepatic stellate cells are involved in ECM remodeling mediated by the secretion of matrix metalloproteinases and activation of natural killer (NK) cells, which clear the senescent cells. Recently, Grosse et al. developed *p16-Cre* and *p16-CreERT2* knock-in mice to perform genetic lineage tracing of *p16*^*INK4a*^-positive cells; they found that the p16^INK4a^-positive senescent cells that increase in the liver with aging are mainly liver sinusoidal endothelial cells (LSECs) [78]. Although the authors hypothesized that the specific elimination of p16^INK4a^-positive LSECs would enhance the regenerative function of the liver, they found that eliminating p16^INK4a^-positive LSECs in fact promoted liver fibrosis in the perivascular areas and caused thrombocytopenia [78]. In addition to the liver, cellular senescence in the heart and skeletal muscles, especially in fibroblast mesenchymal cells [49, 95, 97], has been shown to potentially suppress tissue fibrosis.

## Transient or persistent?

Although it is difficult to clearly distinguish between beneficial and detrimental effects of cellular senescence, beneficial effects are apparently characterized by a transient increase in the early phases of tissue damage followed by immediate clearance, whereas detrimental senescence is characterized by persistent accumulation after tissue damage. One leading study by Demaria et al. showed that a transient increase in senescent fibroblasts promotes wound healing [4]. Recently, PDGFRα-positive mesenchymal cells in subcutaneous adipose tissue were found to undergo senescence and contribute to wound healing in healthy mice in addition to dermal fibroblast senescence [110]. The research findings indicated that senescent PDGFRα-positive mesenchymal cells in subcutaneous adipose tissue increased 2 days after injury and then decreased by 8 days post-injury. However, in diabetic mice, mesenchymal cells delayed the onset of senescence following injury and accumulated in the wound region with SASP expression, including IL-11 and CCL11, thereby hindering healing [110]. Furthermore, senescent PDGFRα-positive cells in the subcutaneous adipose tissue of non-diabetic patients decreased with time after wounding, whereas they increased with time in patients with diabetes, suggesting that the persistent accumulation of senescent cells promotes the deterioration of diabetic wounds [110]. Other studies have supported the beneficial effects of transient cellular senescence and reported neonatal cardiac and muscle regeneration [95, 98].

Immune cells, especially macrophages, cytotoxic T cells, and NK cells are involved in regulating the clearance and accumulation of senescent cells. Sturmlechner et al. reported that p21 ^WAF1/Cip1^ not only maintains the cell cycle arrest of senescent cells but also mobilizes macrophages by secreting CXCL14 as a p21-activated secretory phenotype, polarizing them into M1-type macrophages, and inducing a system by which senescent cells are eliminated by cytotoxic T cell recruitment [111]. Although the p21-activated secretory phenotype does not recruit NK cells, another study reported that senescent cells could activate NK cells and eliminate senescent cells by increasing the expression levels of UL16 Binding Protein 2 (ULBP2) and IL-8, which are ligands for the NK cell receptor NKG2D [109]. Other studies have shown that transplantation of senescent mesenchymal cells into a mouse model of chronic myositis increases M1-type macrophages and NK cells and promotes tissue remodeling, whereas transplantation of *Cdkn2a*-KO mesenchymal cells does not increase their abundance [49]. More recently, studies using both diabetic retinopathy patients and mouse models of ischemic retinopathy have shown that the SASP in senescent vascular endothelial cells stimulates the attraction of neutrophils and the production of neutrophil extracellular traps, both of which induce senescent cell apoptosis and their removal, consequently promoting the pruning and remodeling of pathological neovascular vessels [112]. Thus, senescent cells have a system by which they can be removed by activating immune cells, thereby inhibiting their accumulation in tissues and organs; conversely, immune-escaping senescent cells have also been confirmed.

Previous research has demonstrated that CD47, also known as the “don’t eat me” signal, is increased in senescent cells to prevent phagocytosis by macrophages [113]. Similarly, PD-L1, also known as the “don’t find me” signal, is upregulated in senescent cells, helping them to evade attack by cytotoxic T cells [114]. HLA-E, a non-classical MHC class I molecule, is increased in senescent cells, allowing them to evade attack by both cytotoxic T and NK cells [115]. Collectively, these mechanisms attenuate the immune responses of macrophages, cytotoxic T cells, and NK cells against senescent cells.

## Conclusions

Senescent cells are potential therapeutic targets because of their involvement in chronic inflammation, fibrosis, and aging. However, cellular senescence also possesses beneficial cellular functions, such as promoting tissue regeneration/repair, inhibiting fibrosis, and facilitating reprogramming. The precise mechanisms governing this complex aspect of cellular senescence are yet to be fully elucidated. Among the various factors that influence cellular senescence, “time” appears to play a significant regulatory role.

While the transient or persistent presence of senescent cells is important to the type of senescence, further studies are necessary to elucidate the systemic and cellular differences between these two phenotypes, which still have not been fully characterized. Unraveling new pathways associated with these phenotypes may realize the development and identification of new drugs, biomarkers, or therapeutic targets. In addition, because senescence has apparent dual roles, one outstanding question is how beneficial effects of cellular senescence can be preferentially promoted. Future studies should investigate how the transient, beneficial form of cellular senescence can be promoted, thereby allowing better treatment of various diseases without the associated health issues of aging. In conclusion, investigating various aspects of senescent cells, both beneficial and detrimental, is crucial for the development of safe and effective disease prevention and treatment strategies targeting these cells.

## Data Availability

Not applicable.

## Abbreviations

SASP: Senescence-associated secretory phenotype
SA-β-gal: Senescence-associated-β-galactosidase
ROS: Reactive oxygen species
RS: Replicative senescence
OIS: Oncogene-induced senescence
TIS: Therapy-induced senescence
SIS: Stress-induced senescence
MiDAS: Mitochondria dysfunction-induced senescence
IIS: Immunologically-induced senescence
DDR: DNA damage response
γH2AX: Phosphorylating histone H2AX
MDC1: Mediator of DNA damage checkpoint 1
53BP1: p53 binding protein 1
ATM: Ataxia telangiectasia mutated
ATR: ATM and Rad3-related
CHK: Checkpoint kinase
CDK: Cyclin-dependent kinase
RB: Retinoblastoma
NF-κB: Nuclear factor kappa-light-chain-enhancer of activated B cells
p38MAPK: p38 mitogen-activated protein kinase
mTOR: Mammalian target of rapamycin
C/EBPβ: CCAAT-enhancer-binding protein β
cGAS: cyclic GMP–AMP synthase
STING: Stimulator of interferon genes
IL: Interleukin
CCL: C–C motif chemokine ligand
CXCL: C-X-C motif chemokine ligand
TGF-β: Transforming growth factor-β
EGF: Epidermal growth factor
GDF: Growth differentiation factor
MMP: Metallopeptidase
PCNA: Proliferating cell nuclear antigen
CyTOF: Mass cytometry by time-of-flight
SBB: Sudan Black B
HSV-TK: Herpes simplex virus-1 thymidine kinase
GCV: Ganciclovir
NASH: Nonalcoholic steatohepatitis
SCAPs: Senescent cell anti-apoptotic pathways
BCL: B-cell/CLL lymphoma
PI3K: Phosphoinositide 3-kinase
HIF: Hypoxia inducible factor
HSP: Heat shock protein
DMD: Duchenne muscular dystrophy
iPSC: induced pluripotent stem cell
ECM: Extracellular matrix
CCN1: Cellular communication network factor
PDGFRα: Platelet-derived growth factor receptor alpha
FAPs: Fibro/adipogenic progenitors
BaCl2: Barium chloride
PD-L1: Programmed cell death ligand 1
AMPK: AMP-activated protein kinase
EREG: Epiregulin
BRAF: B-Raf proto-oncogene, serine/threonine kinase
SAHFs: Senescence-related heterochromatin foci
CCl_4_: Carbon tetrachloride
NK: Natural killer
LSECs: Liver sinusoidal endothelial cells
ULBP2: UL16 Binding Protein 2
